# Knowledge of cataracts and eye care utilization among adults aged 50 and above in rural Western China

**DOI:** 10.3389/fpubh.2022.1034314

**Published:** 2022-11-15

**Authors:** Kang Du, Hongyu Guan, Yunyun Zhang, Yuxiu Ding, Decai Wang

**Affiliations:** ^1^College of Economics, Xi'an University of Finance and Economics, Xi'an, China; ^2^Center for Experimental Economics in Education, Shaanxi Normal University, Xi'an, China; ^3^Zhongshan Ophthalmic Center, State Key Laboratory of Ophthalmology, Sun Yat-sen University, Guangzhou, China

**Keywords:** cataract, cataract knowledge, eye care utilization, rural China, elder

## Abstract

**Background:**

Cataracts are highly prevalent in rural China, but patient utilization of eye care services is low. Cataract knowledge is regarded as an important factor in eye care utilization. Few studies, however, have been conducted to measure the level of cataract knowledge and its relationship with eye care utilization among older adults in rural China. Hence, we described cataract knowledge and investigated the relationships between cataract knowledge and eye care utilization among adults (≥50 years) in rural Western China.

**Methods:**

A large community-based cross-sectional study was conducted from October to December 2020 among adults aged 50 years and older in 73 rural villages in Qingcheng County, Western China. The participants underwent an eye examination at their village clinic to determine whether they had cataracts. Participants also answered a questionnaire about cataracts, eye care utilization status, and sociodemographic characteristics. The collected data underwent descriptive and multiple logistic regression analyses.

**Results:**

The eye examinations revealed that 45.15% (675/1,495) of participants had cataracts in at least one eye, yet 90.67% (612/675) were left untreated. The average score achieved by participants about cataract knowledge was 4.91 points (total score was eight points). Correct knowledge about cataracts was positively associated with cataract screening and cataract surgery. Elders with better cataract knowledge were 2.21 times more likely to undergo cataract screening (OR, 2.21; 95% CI, 1.54–3.16) and 5.61 times more likely to undergo cataract surgery (OR, 5.61; 95% CI, 2.87–10.98). More than half had three fundamental misconceptions about how cataracts should be treated, how much they should cost, and when they should be treated. Participants who did not have these misconceptions were more likely to receive cataract screening by 1.21–2.02 times and cataract surgery by 1.76–3.48 times.

**Conclusion:**

There are misunderstandings about cataract treatment methods, treatment costs and timing in the sample areas. A better understanding of cataracts was significantly positively associated with cataract screening and cataract surgery. Health education on cataracts and regular eye examinations are needed to improve eye care utilization in rural China.

## Introduction

Cataract remains the leading cause of blindness in developing countries (1). The prevalence of cataracts is highly correlated with age and more prevalent in adults over 50 years old (2). If left untreated, cataracts worsen vision and reduce the quality of life by limiting the patient's productive activities and lowering personal and family economic levels (3, 4). People with cataracts are also more prone to depression and social isolation (5). Some studies have also found that cataracts pose other risks, such as increased falls, a common cause of death in older adults (6, 7).

China provides a representative case for cataract study. Cataracts caused 18.14 million Chinese people to have a moderate or severe visual impairment in 2019, and 2.95 million became blind (8). The disease burden caused by cataracts in China ranks second globally, only lower than in India (9). As the aging population grows in China (10), timely cataract treatment is necessary.

Surgery is the only effective way to treat cataracts and has been rated by the WHO as one of the most cost-effective medical procedures (2). In addition, with the development of surgical technology, surgical vision can be recovered quickly, and the visual effect is good (1). However, China's cataract surgical rate (CSR) is low, especially in rural areas. Cataract Surgical Rate, which refers to the number of Cataract surgery each year for every million people, is the most commonly used national standard. The National Health Commission reported in June 2020 that the CSR in China was close to 3,000 (11), which is far lower than the average rate of 6,000 CSR in developing countries, and 10,000 CSR in developed countries average rates (12). CSR saw a large urban-rural gap in China. An empirical study points out that in 2012, the CSR in Shanghai's urban area exceeded 6,000, while is only 500 in rural areas (13). In addition to the CSR indicator, cataract surgical coverage (CSC) provides a more accurate measure of how many patients who require cataract surgery have already had cataract surgery (4). A population-based sample representative of rural areas throughout China finds that CSC among those with VA worse than 20/200 in both eyes because of cataracts was 62.8% (14), which has not met the World Health Organization's minimum CSC standard (85%).

The main barrier to the cataract surgery rate may come from the demand side, especially patients' knowledge of cataracts (15, 16). In China, villagers can reduce at least 50% cost of cataract surgery through the New Rural Cooperative Medical System (17). On the other hand, many studies find that awareness and knowledge of cataracts is the main barrier to the low cataract surgery rate (15, 18, 19). A study in southern China found that only 10% of people know adequately about cataracts (20).

Studies suggested that individual awareness and knowledge of eye diseases are important factors in screening, diagnosis, treatment compliance, and prevention (21). Furthermore, assessing cataract knowledge is a prerequisite for designing cataract health education interventions (22, 23). However, previous studies were conducted in cities or areas with better economic levels (16, 22, 24) and were small samples (19, 20, 25). Besides, studies targeting the population over 50 years of age are insufficient. Nevertheless, paying attention to the cataract knowledge level of this group of people is essential for the timely treatment of cataracts. More research was needed in this field. Therefore, this paper aims to describe the knowledge of cataracts among adults aged 50 and above in rural Western China and to analyze the relationships between knowledge of cataracts and eye care utilization using a large-scale community-based sample.

## Methods

### Setting

A community-based, cross-sectional study was conducted among adults aged 50 and above in rural areas of Qingchen county, Gansu Province, Northwestern China. This study was carried out from October to December 2020. Qingcheng County is located 60 km from the main Qingyang City. Two public hospitals provide different eye care services in Qingcheng, including cataract screening and cataract surgery. Five hospitals in Qingyang city also provide cataract treatment services.

Qingcheng County is in the eastern part of Gansu Province. Gansu province is relatively underdeveloped compared to other regions of China. Official statistics indicate that in 2020, villagers' per capita income in Gansu was 39.6% lower than the national level. The villagers' per capita income in Qingcheng County was 2.5% lower than in Gansu province. Our sample area is more representative of the situation in poorer rural areas.

### Sampling

The study sample was collected using a random sampling procedure at the village level. First, a list of the population in each village was obtained through the county people's hospital, involving 153 villages. Ten villages were dropped due to the total population of the village being less than 800. Then, we randomly selected half of the 143 villages as sample villages. Hence, 73 villages were included in this study. Finally, all residents who were 50 years old and above in these villages participated in this study.

Eligibility criteria included being a registered resident of Qingcheng, aged 50 years or older, able to give oral informed consent and verbally answer questions on the researcher-administered questionnaire. A total of 1,554 participants were eligible for the study, among which 1,495 provided complete information required by the questionnaire. The response rate was 96.2%.

### Data collection

#### Eye examinations

The survey included two parts: eye examinations and a questionnaire survey. The eye examinations were carried out by a team of five people, including an ophthalmologist and a nurse from the local public hospital, an experienced ophthalmologist from the Zhongshan Ophthalmic Center, and two questionnaires.

Three days before the eye examinations, the village clinic doctor informed the villagers aged over 50 to attend the eye examinations on the appointed day and informed them that the eye examinations were free. The eye examinations session lasted from 9 a.m. to 5 p.m. at the village clinic, during which the ophthalmic screening team offered free eye examinations to all villagers aged above 50. Eye examinations included two steps: vision screening and cataract diagnosis. First, the nurse measured participants' presenting visual acuity (PVA) by Early Treatment Diabetic Retinopathy Study (ETDRS) charts, a worldwide standard instrument for assessing visual acuity (26). If one's PVA was ≤0.3 in the better eye (a recommended criteria for cataract surgery) (27), he/she would proceed to the cataract screening. Secondly, the ophthalmologist conducted a full ocular examination of pupil dilation using a slit lamp microscope to diagnose the cataract. When there is a dispute about cataract diagnosis, the result is subject to the ophthalmologist from the Zhongshan Ophthalmic Center. Visual acuity and further cataract diagnosis results were recorded by the nurse and the ophthalmologist, respectively.

### Questionnaire survey

The questionnaire survey was executed in the form of a face-to-face interview. The questionnaire survey consists of three parts: basic information, eye care utilization and knowledge of cataracts. Basic information included age, gender, education status and marital status. The facts, such as whether the interviewee lives with at least one child or not and the annual household income, were also included in the information.

Eye care utilization can be measured by asking participants to answer yes or no to (1) whether they had at least a vision screening in the past, (2) whether they ever had cataract screening in the past, and (3) whether they were ever had cataract surgery. According to these answers, eye care utilization was defined as three dummy variables (0 = No, 1 = Yes).

Participants' knowledge of cataracts was assessed using eight questions in our study. Participants' knowledge of cataracts was assessed using eight questions in our study. Following the literature (15, 20), these questions were developed by a group of health experts from Shaanxi Normal University and Zhongshan Ophthalmic Center (an authoritative ophthalmology institution in China). Each item was equally weighted. The eight dichotomous variables (knowledge of cataracts) were given a score of 0 (wrong answer) or 1 (right answer).

Hence, the total score for all knowledge questions ranged from 0 to 8 points. We also generate a dummy variable named cataract knowledge level, which takes the value of 1 if the total score is higher than the mean (good knowledge), and 0 (poor knowledge).

### Ethical consideration

The study was conducted following the Declaration of Helsinki and approved by the Stanford University Institutional Review Board (ID. 64279). Health education about cataracts was given after the survey for each study participant. We obtained the respondents' oral informed consent; All data were analyzed anonymously.

### Statistical analysis

Frequencies and percentages were used to describe data. Multivariate logistic analyses were used to analyze the associations between cataract knowledge and eye care utilization. Covariables were included in the regression, including age, gender, education status, marital status, whether the interviewee lives with at least one child, household income and whether PVA ≤0.3 in either eye. This study's result was deemed statistically significant if the *P*-value was < 0.05. Odds ratios (OR), 95% confidence intervals (CI), and *P*-value are presented in **Tables 4**, **5**. All analyses were conducted using Stata15.1 (Stata Corp).

## Results

### Sociodemographic characteristics of the sample

Among 1,554 adults aged ≥50 years, 1,495 of them completed the interview. The response rate was 96.20%. The mean age was 63 years (±9 years). Among the study participants, about 60% are female. In terms of education, 43.75% of respondents had no formal education. Three-quarters (1,124/1,495) of the study participants have a spouse, and more than half (877/1,495) do not live with a child. Forty-six percent (690/1,495) of the study participants' household income was <10,000 RMB (Criteria for low-income families in rural China). Most respondents (1,124/1,495) have PVA less than or equal to 0.3 in either eye (Table 1).

**Table 1:** Sociodemographic characteristics of adults aged 50 and above in rural Western China (*n* = 1,495).

**Variables**	**Frequency**	**Percent (%)**
Age		
50–59	641	42.88
60–69	519	34.72
70 & above	335	22.41
Gender		
Male	609	40.74
Female	886	59.26
Education status		
No formal education	654	43.75
Primary education	496	33.18
Secondary education and above	345	23.08
Marital status		
Don't have a spouse	371	24.82
Have a spouse	1,124	75.18
Live with a least one child		
No	877	58.66
Yes	618	41.34
Household Income (RMB)		
<10,000	690	46.15
10,000–20,000	487	32.58
≥20,000	318	21.27
Presenting visual acuity ≤ 0.3 in either eye		
No	964	64.48
Yes	531	35.52

### History of eye care utilization and prevalence of cataract

Before this study, 35.52% of the 1,495 participants had received vision screening and 17.99% had undergone cataract screening. Our vision and cataract screening revealed that 45.15% of participants had cataracts in at least one eye (Table 2). Among those diagnosed with cataracts, the majority (90.67%) of them were left untreated, suggesting the cataract surgical coverage was <10% in our sample, lower than the World Health Organization's minimum cataract surgery coverage standard (85%).

**Table 2:** Previous history of eye care utilization and prevalence of cataracts of adults aged 50 and above in rural Western China (*n* = 1,495).

**Variables**	**Frequency**	**Percent (%)**
Previous vision screening		
Yes	531	35.52
No	964	64.48
Previous cataract screening		
Yes	269	17.99
No	1,226	82.01
Diagnosed with cataracts by this study		
Yes	675	45.15
No	820	54.85
Had a cataract surgery		
Yes	63	9.33
No	612	90.67

### Participants' knowledge of cataracts

Table 3 shows the knowledge of cataracts among the participants presented in the correct rate of questions from high to low. The participants' mean (±SD) knowledge score is 4.91 ± 1.91 points. Among the eight questions, the correct rate of five questions is more than 50%. As for the level of cataract knowledge, we find nearly two-thirds (62.88%) of the participants had good knowledge of cataracts.

**Table 3:** Knowledge about cataracts among adults aged 50 and above in rural Western China (*n* = 1,495).

**Questions**	**Scoring scheme**	**Correctly answered**	
		***N***	**%**
1. Whether the elders need to do regular cataract examinations?	Yes = 1 mark	1,226	82.01
2. Can cataracts be cured by surgery?	Yes = 1 mark	1,087	72.71
3. Whether cataract surgery is safe?	Yes = 1 mark	1,060	70.90
4. Whether cataracts are a common disease for elders?	Yes = 1 mark	1,034	69.22
5. Will cataract surgery improve eyesight?	Yes = 1 mark	1,015	67.89
6. Can cataracts be cured with medications?	No = 1 mark	714	47.76
7. Whether cataract surgery is covered by New Cooperative Medical System?	Yes = 1 mark	663	44.35
8. Should a patient delay cataract surgery before being blind?	No = 1 mark	540	36.12

Specifically, the accuracy of each question is ranked in descending order as follows. The importance of regular cataract examinations was acknowledged by 1,226 (82.01%) participants. About 70% of the participants believed that cataracts could be treated with surgery and that the surgery was safe. It was understood by 1,034 participants (69.22%) that cataracts are a common disease for older. More than two-thirds (67,89%) believed that cataract surgery improves visual acuity. However, the accuracy of the three questions is < 50%. Over half of the participants had the wrong idea about how to treat cataracts, as evidenced by the 714 (47.76%) people who correctly answered that cataracts cannot be cured with medications. Less than half of those participants (44.35%) knew that the new cooperative health system covered cataract surgery. In addition, only 37 (2.47%) participants know the specific reimbursement rate of cataract surgery. As for the question with the lowest accuracy, only 540 (36.12%) participants realized that cataracts should be treated in time, which means that about two-thirds of the participants might delay cataract surgery before losing eye vision (Table 3).

### Knowledge of cataracts and eye care utilization

Table 4 shows the associations of cataract knowledge level with eye care utilization (cataract screening and surgery) estimated by multiple logit models. We find that cataract knowledge level was significantly associated with cataract screening and cataract surgery. Participants with good cataract knowledge were 2.21 times more likely to undergo cataract screening (OR = 2.21; 95% CI = 1.54–3.16, *P* = 0.007). Moreover, cataract patients with good knowledge were 4.92 times more likely to undergo cataract surgery (OR = 4.92; 95% CI = 2.50–9.70, *P* = 0.001).

**Table 4:** Multivariate estimation of the association of knowledge level about cataracts with eye care utilization.

**Variables**	**Cataract screening (*****n*** = **1,495)**		**Cataract surgery (*****n*** = **675)**	
	**OR (95%CI)**	***P*-value**	**OR (95%CI)**	***P*-value**
Cataract knowledge level				
Poor	1	–	1	–
Good	2.21 (1.54–3.16)	0.007	4.92 (2.50–9.70)	0.001
Age				
50–59	1	–	1	–
60–69	2.46 (1.67–3.62)	0.000	1.64 (0.58–4.70)	0.353
70 & above	2.60 (1.66–4.06)	0.000	3.51 (1.69–7.27)	0.095
Gender				
Male	1	–	1	–
Female	1.52 (1.09–2.12)	0.019	0.72 (0.44–1.18)	0.195
Education status				
No formal education	1	–	1	–
Primary education	0.97 (0.66–1.44)	0.937	1.08 (0.60–1.95)	0.794
Secondary education and above	1.34 (0.87–2.07)	0.070	1.04 (0.55–1.97)	0.899
Marital status				
Don't have a spouse	1	–	1	–
Have a spouse	0.81 (0.57–1.15)	0.290	0.61 (0.38–0.96)	0.032
Live with a least one child				
No	1	–	1	–
Yes	0.97 (0.70–1.34)	0.867	0.93 (0.59–1.49)	0.771
Household income (RMB)				
<10,000	1	–	1	–
10,000–20,000	0.69 (0.47–1.00)	0.075	0.61 (0.35–1.08)	0.090
≥20,000	1.03 (0.68–1.56)	0.858	1.45 (0.83–2.56)	0.189
Presenting visual acuity≤0.3 in either eye				
No	1	–	1	–
Yes	2.46 (1.80–3.37)	0.000	2.69 (1.68–4.29)	0.000

Several sociodemographic characteristics were also associated with cataract screening and cataract surgery (Table 4). Older age (*P* < 0.001); female (*P* < 0.05), and PVA ≤0.3 in either eye (*P* < 0.001) were associated with cataract screening, while education status, marital status, living status and household income were not; As for the cataract surgery, the following characteristics were associated with cataract surgery: don't have a spouse (*P* < 0.05) and PVA ≤0.3 in either eye (*P* < 0.001).

Furthermore, we investigated the association between these eight categories of knowledge and eye care utilization respectively to investigate which category of knowledge influences the participants' eye care utilization. The results are shown in Table 5. In general, participants with adequate knowledge of cataract surgery were more likely to undergo cataract screening and surgery.

**Table 5:** Multivariate estimation of the association of knowledge about cataracts with eye care utilization^a^.

**Variables**	**Cataract screening (*****n*** = **1,495)**		**Cataract surgery (*****n*** = **675)**	
	**OR (95% CI)**	***P*-value**	**OR (95% CI)**	***P*-value**
1. Whether the elders need to do regular cataract examinations?	1.44 (0.94–2.21)	0.092	1.63 (0.79–3.35)	0.185
2. Can cataracts be cured by surgery?	1.64 (1.15–2.35)	0.000	4.08 (1.84–9.08)	0.001
3. Whether cataract surgery is safe?	1.49 (1.05–2.11)	0.025	9.04 (3.30–24.79)	0.000
4. Whether cataracts are a common disease for elders?	1.46 (1.03–2.08)	0.036	1.62 (0.88–2.99)	0.119
5. Will cataract surgery improve eyesight?	1.99 (1.40–2.83)	0.000	5.59 (2.63–11.89)	0.000
6. Can cataracts be cured with medications?	2.02 (1.48–2.76)	0.000	3.24 (1.86–5.65)	0.000
7. Whether cataract surgery is covered by New Cooperative Medical System?	1.44 (0.96–2.16)	0.074	1.20 (0.72–2.03)	0.484
8. Should a patient delay cataract surgery before being blind?	1.21 (0.86–1.68)	0.271	1.82 (1.05–3.16)	0.032

a The answer “Wrong” is the comparison in each regression.

For cataract screening, participants who correctly answered the question “cataracts cannot be cured with medications” were two times more likely to undergo cataract screening (OR = 2.02; 95% CI =1.48–2.76), which has the strongest influence on the screening among eight questions. Participants who realized that “cataracts can be cured by surgery,” “cataracts surgery is safe,” “cataracts are a common disease,” and “cataract surgery can improve eyesight” were more likely to undergo cataract screening than their counterparts. However, the knowledge of “Should a patient delay cataract surgery before being blind?” was insignificantly associated with cataract screening (*P* = 0.271). It is understandable, considering that this knowledge seems to be indirectly related to accepting cataract screening.

For cataract surgery, patients who realize that “cataracts can be cured by surgery,” “cataracts surgery is safe,” “cataracts cannot be cured with medication,” “cataract surgery can improve eyesight” and “a patient should not delay cataract surgery before being blind” were more likely to undergo cataract surgery than their counterparts.

## Discussion

Like in other countries or areas, cataracts in rural Western China remain a devastating condition. Cataracts were diagnosed in 45.15% of the participants aged 50 and above, which is consistent with earlier research (8, 28). However, our study found that the rate of eye care utilization in rural areas was much lower than in urban or developed areas. According to the American Academy of Ophthalmology's (AAO) guidelines, people aged 65 years and older should have a vision test every 1–2 years (29). Whereas, only one-third of the subjects (35.52%) in this study reported they had sought vision screening in the past, and even fewer (17.99%) indicated they had undergone cataract screening. Among the patients diagnosed with cataracts, only 9.33% underwent cataract surgery. This proportion was only half of that in urban areas (17.81%) (25). This finding implies that improving cataract surgical coverage in rural areas of China continues to be challenging.

The overall cataract knowledge level found in the current study was 62.88%, which is close to a study done in northwest Ethiopia (61.7%) (19) but lower than studies done in Chengdu, China (70.8%) (24) and Nepal (70.4%) (30). The target population for the last two studies was age ≥40. Hence, these subtle differences may be due to the difference in the target population and study setting.

Specifically, in our sample, adults over 50 years old are unable to understand cataract knowledge mainly in 3 aspects. The first and biggest misconception is that many people believe that cataracts need to be treated until they are blind, which means villagers do not know the right time to treat cataracts. On the second, more than half of the participants were unaware that new rural cooperative medical care reimburses the cost of cataract surgery, and even fewer were aware of specific reimbursement ratios. As a result, these individuals may choose not to seek eye care services because of miscalculating treatment costs for their cataracts. While on the third, 52.24% of the participants thought that taking medicine could cure cataracts, which illustrated that the correct treatment modality for these individuals was unclear. People with this misconception may choose not to have cataract surgery because they think medications (e.g., eye drops) can replace surgery. This result was also demonstrated by the multiple regression results in Table 5 (Row 6, columns 3), in which individuals who believed that cataracts could not be treated with medication were 3.24 times more likely to have surgery than those with these misconceptions. These results show that the correction of misunderstandings above must be covered in the health education on cataracts. Of course, other cataract-related knowledge in health education is also needed.

Furthermore, we found that those with better knowledge of cataracts were more likely to participate in cataract screening and surgery. Whether cataract knowledge is analyzed as a whole or divided into eight questions, the results are robust. The results have also been found in other related studies (15, 31, 32). This study enlightens that education on cataract knowledge in rural areas may help improve eye care utilization. In addition to cataract knowledge, we also found that age and vision status may be major factors affecting eye health services.

Our study still has several limitations. Firstly, our results may be skewed because villagers with an existing vision impairment or cataract would be more likely to participate than those with no such problem. However, the prevalence of cataracts in this study is generally consistent with previous studies suggesting that any bias would be small. Further studies are needed to evaluate the causal relationship between knowledge of cataracts and eye care utilization. Second, the utilization of eye care services are self-reported variables, and self-report bias may influence our results. Additionally, this study describes a population from a single region in rural Western China. Thus, extending our results to other settings must be done only with caution.

Despite its limitations, based on a large sample, our study provides new data on the villagers' knowledge of cataracts and its relationship with eye care utilization in rural areas. Moreover, the study has described the key misunderstanding in cataract cognition in rural areas and generated a useful prerequisite for eye health education interventions in the future. Therefore, the results of this study will provide researchers, policymakers, and resource distributors with basic information to plan health education and promotion programs that enable early cataract prevention and treatment options.

## Conclusions

Cataract patients in Western China's rural areas have low utilization of eye care and misconceptions about cataract treatment methods, timing, and cost. Better knowledge of cataracts was significantly and positively associated with cataract screening and surgery. With the aging population in the future, the scale of cataract patients and the number of blindness caused by cataracts will continue to expand. Hence, it is recommended for the national and regional ministry of health offices to organize different health education programs focusing on regular eye examinations in rural areas.

## Data availability statement

The raw data supporting the conclusions of this study is available from the corresponding author upon reasonable request.

## Ethics statement

The study was conducted following the Declaration of Helsinki and was reviewed and approved by the Stanford University Institutional Review Board (ID. 64279). Written informed consent for participation was not required for this study in accordance with the national legislation and the institutional requirements.

## Author contributions

HG conceptualized and supervised the study. KD developed the proposal, did the data collection, analyzed the data, and wrote the manuscript. YZ, YD, and DW contributed to the study design, made substantial contributions to the acquisition, and quality assurance of the data. All authors read and approved the final manuscript.

## Funding

This study was funded by Sany Foundation (Beijing, China). The authors are also supported by Higher Education Discipline Innovation Project, Grant Number B16031. The study's funder had no role in study design, data collection, data analysis, data interpretation, or writing of the report.

## Conflict of interest

The authors declare that the research was conducted in the absence of any commercial or financial relationships that could be construed as a potential conflict of interest.

## Publisher's note

All claims expressed in this article are solely those of the authors and do not necessarily represent those of their affiliated organizations, or those of the publisher, the editors and the reviewers. Any product that may be evaluated in this article, or claim that may be made by its manufacturer, is not guaranteed or endorsed by the publisher.
